# Adaptive coding of reward in schizophrenia, its change over time and relationship to apathy

**DOI:** 10.1093/brain/awae112

**Published:** 2024-04-12

**Authors:** Mariia Kaliuzhna, Fabien Carruzzo, Noémie Kuenzi, Philippe N Tobler, Matthias Kirschner, Tal Geffen, Teresa Katthagen, Kerem Böge, Marco M Zierhut, Florian Schlagenhauf, Stefan Kaiser

**Affiliations:** Clinical and Experimental Psychopathology Laboratory, Department of Psychiatry, University of Geneva, 1205 Geneva, Switzerland; Clinical and Experimental Psychopathology Laboratory, Department of Psychiatry, University of Geneva, 1205 Geneva, Switzerland; Department of Psychiatry, Geneva University Hospitals, 1205 Geneva, Switzerland; Laboratory for Social and Neural Systems Research, Department of Economics, University of Zurich, 8006 Zurich, Switzerland; Department of Psychiatry, Geneva University Hospitals, 1205 Geneva, Switzerland; Department of Psychiatry and Psychotherapy, Charité—Universitätsmedizin Berlin, 10117 Berlin, Germany; Department of Psychiatry and Psychotherapy, Charité—Universitätsmedizin Berlin, 10117 Berlin, Germany; Department of Psychiatry and Psychotherapy, Charité—Universitätsmedizin Berlin, 10117 Berlin, Germany; Department of Psychiatry and Psychotherapy, Charité—Universitätsmedizin Berlin, 10117 Berlin, Germany; Department of Psychiatry and Psychotherapy, Charité—Universitätsmedizin Berlin, 10117 Berlin, Germany; Department of Psychiatry, Geneva University Hospitals, 1205 Geneva, Switzerland

**Keywords:** adaptive coding, schizophrenia, reward, functional MRI, monetary incentive delay task, negative symptoms

## Abstract

Adaptive coding of reward is the process by which neurons adapt their response to the context of available compensations. Higher rewards lead to a stronger brain response, but the increase of the response depends on the range of available rewards. A steeper increase is observed in a narrow range and a more gradual slope in a wider range. In schizophrenia, adaptive coding appears to be affected in different domains, especially in the reward domain. Here, we tested adaptive coding of reward in a large group of patients with schizophrenia (*n* = 86) and control subjects (*n* = 66). We assessed: (i) the association between adaptive coding deficits and symptoms; (ii) the longitudinal stability of deficits (the same task was performed 3 months apart); and (iii) the stability of results between two experimental sites.

We used functional MRI and the monetary incentive delay task to assess adaptation of participants to two different reward ranges: a narrow range and a wide range. We used a region-of-interest analysis to evaluate adaptation within striatal and visual regions. Patients and control subjects underwent a full demographic and clinical assessment.

We found reduced adaptive coding in patients, with a decreased slope in the narrow reward range with respect to that of control participants, in striatal but not visual regions. This pattern was observed at both research sites. Upon retesting, patients increased their narrow-range slopes, showing improved adaptive coding, whereas control subjects slightly reduced them. At retesting, patients with overly steep slopes in the narrow range also showed higher levels of negative symptoms.

Our data confirm deficits in reward adaptation in schizophrenia and reveal an effect of practice in patients, leading to improvement, with steeper slopes upon retesting. However, in some patients, an excessively steep slope may result in poor discriminability of larger rewards, owing to early saturation of the brain response. Together, the loss of precision of reward representation in new (first exposure, underadaptation) and more familiar (retest, overadaptation) situations might contribute to the multiple motivational symptoms in schizophrenia.

## Teaser

Kaliuzhna *et al.* explore how the brains of patients with schizophrenia adapt to the range of available rewards and how this is related to symptoms of apathy. Adaptive coding is impaired in patients relative to control subjects, with the impairment being driven by a reduced ability to increase brain responses to smaller rewards in a narrow reward range.

## Introduction

**Figure 1:**
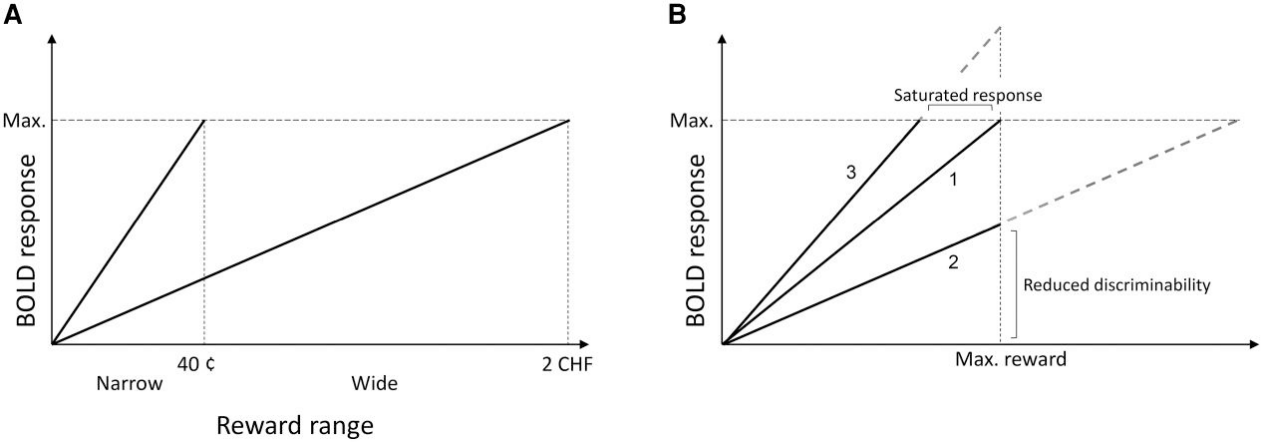
**Schematic representation of range adaptation of the blood oxygenation level-dependent (BOLD) response.** (**A**) Adaptive coding. To encode the unlimited range of possible rewards with a limited coding range efficiently, the brain adapts its response dynamically to the range of most probable rewards in the current monetary incentive delay condition. This entails a shallower slope when more rewards need to be represented. It also entails that the same reward (e.g. 40¢) will result in different activation levels depending on the reward range to which it belongs: a maximal response in the narrow range and a much weaker response the wide range context. (**B**) Forms of inefficient adaptive coding. Efficient adaptive coding of rewards entails a maximal brain response to maximal reward in the current reward context (1), whereas underadaptation (2) would lead to reduced discriminability between available rewards (i.e. different rewards would lead to a very similar brain response). Conversely, overadaptation (3) would result in an incomplete representation of reward options, in particular around the maximal available reward, because a maximal response will be elicited by smaller-than-maximal rewards.

To represent the rewarding value of everyday actions and events adequately, the brain needs to adapt to the context of available rewards. You can be perfectly happy with your salary until you learn that your colleague is earning more. Adaptation is driven by our neuronal systems having a limited capacity to respond but having to represent an unlimited number of potential reward options.^1^ Adaptive neuronal coding is the mechanism by which the firing rate adjusts such that it represents the range (or context) of available stimuli (i.e. range adaptation).^2,3^ This mechanism has been described extensively in different sensory modalities^2,4-6^ and for more complex processes, such as reward valuation.^3,7-10^ Range adaptation is measured as a change in the slope of the neural response depending on the width of the range (Fig. 1A). Given a positive relationship between the behavioural or neural response and the amount of reward, previous work has shown that the slope of the increase of value judgements and the neural signal for increasing rewards differ depending on the range of reward options available.^3,10^ A steeper slope is observed in a narrow range and a shallower slope in a wide reward range, where more options need to be represented, to ensure the most precise discrimination possible between all options.

In terms of neural substrates, the midbrain dopaminergic neurons have been described to adapt their firing rate to the range of the most probable rewards.^11^ In addition, functional MRI (fMRI) work in humans has shown reward adaptation in several regions, including the striatum, the posterior and anterior cingulate cortices, the prefrontal cortex^3^ and the insula.^8,9^

Schizophrenia is a severe mental illness characterized clinically by a diverse set of symptoms. Interestingly, deficits in adaptive processing and integration of contextual information are thought to underlie the visual processing distortions, language and thought disturbances, self-distortions and apathy of patients.^12-14^ In particular, difficulty in the ability to discriminate adequately between rewarding values depending on context might play a role in the motivational negative symptoms of patients,^8,9,15^ which are characterized by a reduction of interest and drive to perform goal-directed actions, leading to diminished engagement in work, leisure and social activities.^16^ Previous work has shown an association between deficient adaptive coding of the reward range predicted by conditioned stimuli and negative symptoms in schizophrenia. At the neural level, adaptive coding in the striatum and the precentral gyrus are inversely related to the psychosis spectrum, being strongest in healthy participants with high schizotypy scores, intermediate in first episode psychosis and weakest in patients with chronic schizophrenia.^9^ Moreover, the reduction in reward adaptation is correlated with negative, positive and global symptom severity [as measured by the Positive and Negative Syndrome Scale (PANSS)].^8,9^ Likewise, Wang *et al*.^10^ have shown reduced range adaptation of reward pleasantness ratings in chronic and first episode patients with schizophrenia. Worse range adaptation in patients was correlated with higher avolition scores in their study (as measured by the Scale for the Assessment of Negative Symptoms (SANS).

What is the nature of reward-adaptive coding deficits in schizophrenia? Although patients appear to increase their brain response with increasing rewards, research shows a reduced difference between the slopes of blood oxygen level-dependent (BOLD) response increase in contexts with a narrow or a wide range of possible rewards.^8-10,15^ This opens several possibilities regarding the specific adaptation deficit. Do patients have difficulty increasing the brain response to rewards in a specific context (e.g. too steep an increase in the wide range or not steep enough in the narrow range)? As shown in Fig. 1B, slopes either too steep or too shallow can lead to an inadequate representation of available reward options. A slope that is too shallow leads to poor discriminability between rewards, because different amounts of reward are associated with similar brain responses. In contrast, a slope that is too steep leads to a lack of discriminability for larger amounts of reward, because the neural response saturates before the maximal available reward levels; thus, any reward larger than the saturation point will have the same neural representation. Our previous work suggested that when comparing a wide with a narrow reward range, patients show selective deficits for the narrow range, demonstrated by a shallower increase in activity than in healthy control subjects.^15^

In the present work, we evaluated reward range adaptation in a large cohort of patients with schizophrenia and healthy control subjects. Our goal was to replicate range adaptation deficits in schizophrenia, and specifically, the nature of this deficit with regard to the wide or the narrow range. Next, we evaluated whether adaptation in patients (and control subjects) is stable over time. Our participants performed the same task twice, 3 months apart, and we assessed whether their performance remained stable across sessions. In addition, we sought to evaluate the relationship between deficits in adaptive coding, and negative symptoms in particular, and the stability of any such association over time in the patients with schizophrenia. Finally, we estimated whether our results are independent of the study site: our participants were recruited in two research centres in Switzerland and Germany.

## Materials and methods

For a complete description of the cohort, refer to the Supplementary material. Note that certain demographic, clinical and experimental values may differ between studies that used the same cohort owing to the specific analyses performed.

### Participants

Patients with schizophrenia were recruited from the University Hospitals of Geneva (out-patient clinics; *n* = 50) and Charité University Hospital of Berlin (in- and outpatient clinics; *n* = 36). Patients were stable according to the treating psychiatrist and had no change in medication in the 2 weeks preceding inclusion in the study. We recruited healthy control subjects from the general population in Geneva (Switzerland; *n* = 31) and Berlin (Germany; *n* = 35) using posters in public spaces and online advertising. Both patients and control subjects were screened with the Mini International Neuropsychiatric Interview (MINI) (for DSM-IV^17^) in order to exclude participants with Axis I disorders. Thus, healthy control subjects were excluded if they or a member of their immediate family had a psychiatric diagnosis (based on self-report by the participants) or if they presented with psychiatric symptoms. Patients were excluded if they presented with florid psychotic symptoms (scores higher than four on the positive subscales of the PANSS). To be included in the study, all participants had to have sufficient French or German language proficiency.

At Session 2 (see ‘Experimental design’ section), several participants dropped out. Thus, in Geneva, 29 control subjects and 39 patients were included, and in Berlin, 26 control subjects and 28 patients. Participants were reimbursed for their participation (20 CHF/h or 10 EUR/h) in addition to the amounts won in the experimental tasks.

### Experimental design

The present experiment was a part of a larger study, in which patients and control subjects came in for two experimental sessions. During the first visit, all participants underwent a detailed clinical assessment (see below) and performed two behavioural protocols. This visit lasted between 2 and 5 h and could be split into two if patients were too tired. Participants came back within 1 week to perform an fMRI session, which included two tasks and anatomical data acquisition. The fMRI session lasted for ∼1.5 h. These two visits corresponded to Session 1. Session 2 was almost identical (it excluded the MINI) and was performed ∼3 months later. It also included two visits, one for the clinical assessment and behavioural protocols and one for the fMRI session. Participants performed the same tasks during both sessions. Owing to the coronavirus disease 2019 pandemic, several Session 2 visits had to be postponed. On average, there were 106.87 [standard deviation (SD) = 22.51] days between Session 1 and Session 2.

### Clinical assessment

All participants underwent a detailed clinical assessment during each of the two visits, conducted in French in Geneva and in German in Berlin. Positive, negative and general symptoms were assessed using the PANSS.^18^ Negative symptoms were assessed specifically using the Brief Negative Symptom Scale (BNSS^19^) and self-assessed using the Self-evaluation of Negative Symptoms (SNS^20^). Extrapyramidal symptoms were assessed using the St-Hans Rating Scale (SHRS).^21^ Depression was assessed with the Calgary Depression Scale for Schizophrenia (CDS).^22^ We assessed general functioning using the Personal and Social Performance scale (PSP)^23^ and the Global Assessment of Functioning scale (GAF).^24^ Cognition was assessed with the Brief Assessment of Cognition in Schizophrenia (BACS).^25^ Assessment at both sites was conducted by psychologists at the doctoral or postdoctoral level and supervised by experienced psychiatrists (the senior authors). Extensive in-person training was conducted with the persons involved in assessment at the beginning of the study, based on video recordings of patients. Given that negative symptoms were the main dimension of interest, inter-rater reliability was assessed for this dimension and was >0.8. Video-based training was also conducted roughly halfway through the study. Both last authors assisted with the assessments punctually and checked the scoring.

### Experimental protocol

Participants performed a variant of the monetary incentive delay task^26^ (Supplementary Fig. 2) twice (i.e. at Session 1 and Session 2). Every trial of this task started with the presentation of a cue indicating the maximal amount that participants could win in this trial (0, 0.40 or 2 CHF in Switzerland and 0, 0.20 or 1 EUR in Germany, to match the socio-economic context of the two countries). Next, participants performed a simple discrimination task, indicating, as fast and correctly as possible, which of the lateral circle stimuli differed from the other two. Finally, feedback informed participants of how much they won on the trial. This last phase was analysed in the present study.

Participants performed a training session outside the scanner (12 trials) and inside the scanner (six trials), followed by two experimental runs (36 trials; ∼6 min each). At the beginning of the experiment, participants were informed that they would receive the total amount won during the experimental runs.

### Data analysis

#### Behavioural analysis

The main outcome of the monetary incentive delay protocol is the difference between reaction times between the three conditions. Participants are faster the higher the amount of reward. Additionally, we also quantified the accuracy of participants and the total amount won in the task.

#### MRI acquisition

In both locations (Campus Biotech in Geneva and Charité Hospital in Berlin), imaging data were collected using a Siemens Magnetom Prisma 3.0 T whole-body scanner. The two machines were equipped with a 64-channel head coil. Functional images were acquired using an echo-planar image sequence with 66 slices acquired in an interleaved fashion, with a multi-band acceleration factor of six. The in-plane resolution was 2 mm × 2 mm, 2 mm slice thickness and a field of view of 224 mm. Volume acquisition had a repetition time (TR) of 1000 ms, an echo time (TE) of 32 ms and a flip angle of 50°. Anatomical data were acquired using a magnetization prepared rapid acquisition gradient (MPRAGE) sequence in 208 sagittal-plane slices of 256 mm × 256 mm with a 1 mm × 1 mm resolution and a slice thickness of 1 mm.

#### Image preprocessing

Preprocessing was run using the fmriprep pipeline.^27^ Smoothing was performed in SPM12 (Statistical Parametric Mapping; Welcome Trust Centre for Neuroimaging, London, UK) using a 5 mm full-width at half-maximum Gaussian kernel. Motion artefacts were detected using the Art toolbox (http://web.mit.edu/swg/software.htm). Scans with head motion of >2 mm and/or changes in mean signal intensity larger than nine were considered outliers and were omitted from the analysis. A total of 1.04% of scans were omitted. The highest percentage of scans omitted in a single participant was 15%.

#### First-level image analysis (within subject)

Following our previous approach,^8,9,15^ to identify the brain regions processing the reward obtained at the end of each trial we used a general linear model with a parametric design. We thus modelled each reward outcome condition (no, small and large reward) separately. The small and large outcome regressors were also modulated parametrically (pmod small reward and pmod large reward, respectively) by the outcome received by each participant in each trial. The small-reward condition thus spans a narrow reward range, and the large-reward condition spans a wide range. The two parametric modulators capture linear deviations from the mean activity induced by the trial-specific reward level and are orthogonal to the mean regressors. We also used several regressors of no interest: a regressor for the anticipation phase (duration between 3.25 and 3.75 s), one for target presentation and one for trials in which participants made an error (modelled at target presentation). Eight regressors were thus used for the first-level analysis. All explanatory variables were convolved with the canonical haemodynamic response function.

#### Second-level image analyses (group comparison)

For the group-level analysis, we interrogated the individual contrast images, which we obtained from the first-level parametric modulators. For this, we used a mask obtained using the same protocol in a large group (*n* = 86) of healthy participants (Giarratana *et al*., unpublished results) at the University of Zurich. The participants in the Zurich study performed the same task, and we used a voxel-wise whole-brain analysis across all participants with a one-tailed *t*-test to interrogate the adaptive coding contrast (the contrast estimates of the large-reward parametric regressor were subtracted from the contrast estimates for the small reward parametric regressor: pmod small reward − pmod large reward), with a statistical threshold of *P* < 0.05, whole-brain voxel-level family-wise error rate corrected for multiple comparisons. We thus defined an independent mask including all regions showing adaptive coding of reward in healthy participants (Supplementary Table2). We took this approach because the adaptive coding response is a subtle variation of the reward response, hence effect sizes will necessarily be smaller than, for example, in a simple comparison between reward and no-reward trials. A targeted region-of-interest (ROI) approach would therefore allow any group differences to be captured better.

In the present sample, within the regions contained in the mask we investigated the adaptive coding contrast (pmod small reward − pmod large reward), in addition to the two conditions separately: the narrow-range reward condition (pmod small reward) and the wide-range reward condition (pmod large reward). We used one-tailed voxel-wise *t*-tests.

We also performed an additional set of analyses using other ROIs. These analyses are reported in the Supplementary material (Supplementary results using a different set of ROIs). The first analysis followed our previous work^8,9^ and interrogated adaptive coding within reward-sensitive regions, obtained from the contrast [(pmod small reward + pmod large reward), at a cluster defining threshold of *P* < 0.0001]. Fourteen ROIs were identified. The second analysis used six striatal ROIs as defined by Mawlawi *et al*.^29^

For all analyses, to assess adaptive coding, in addition to any differences between patients and control subjects, we extracted the mean contrast estimates of our conditions using the Marsbar toolbox.^28^ We compared the groups using ANOVAs on linear mixed effect models.

## Results

### Demographic and clinical data

Demographic data for Sessions 1 and 2 can be found in Tables 1 and 2, respectively. For Session 1, patients with schizophrenia had fewer years of education than healthy control subjects [*F*(1,148) = 19.8, *P* < 0.0001], in addition to lower cognitive scores [BACS total, *F*(1,146) = 69.18, *P* < 0.0001]. Patients in Geneva and Berlin differed only on the parkinsonism score (worse in Geneva, *W* = 485.5, *P* = 0.0001) and the cognition (BACS) score (worse in Geneva, *W* = 1159, *P* = 0.024).

**Table 1:** Demographical and clinical characteristics of patients and control subjects in Session 1

	Session 1			
	Berlin		Geneva	
	SZ (*n* = 36)	HC (*n* = 35)	SZ (*n* = 50)	HC (*n* = 31)
Demographics				
Age, years	38.4 (9.6)	37.8 (7.3)	37.5 (10.2)	38.7 (7.4)
Sex, % male	78	77	80	77
Education, years	13.3 (3.003)	14.8 (2.6)	12.4 (2.8)	14.7 (1.6)
Illness duration, years	13.5 (9.6)	–	12.7 (8.1)	–
Clinical variables				
BNSS				
Apathy	16.1 (9.5)	2.4 (3.8)	13.3 (7.9)	0.5 (0.9)
Diminished expression	7.6 (7.1)	0.8 (1.8)	6.7 (6.6)	0.6 (1.6)
PANSS				
Negative	16.9 (7.3)	8.2 (2.2)	15.9 (6.2)	7.5 (0.9)
Positive	12.4 (5.3)	7.9 (1.7)	10.8 (3.8)	7.03 (0.2)
Total	56.5 (19.3)	33.6 (5.5)	48.5 (10.1)	31.2 (1.9)
SNS				
Apathy	7.7 (4.7)	2.7 (2.7)	8.1 (4.9)	2.8 (2.5)
Diminished expression	7.2 (4.1)	2.7 (2.3)	7.1 (3.2)	2.4 (2.4)
Total	14.97 (8.2)	5.3 (3.9)	14.98 (7.4)	5.2 (4.1)
CDS, total	3.9 (4.5)	0.8 (1.3)	2.4 (2.8)	0.3 (0.7)
BACS, total, *Z*-score	−1.1 (1.4)	1.01 (3.7)	−1.6 (1.1)	0.6 (1.01)
PSP, total	56.3 (19.7)	86.4 (12.04)	55.96 (14.3)	92.7 (4.01)
SHRS, parkinsonism score	1.3 (3.3)	–	2.7 (3.7)	–
Risperidone equivalent	5.6 (4.3)	–	4.8 (2.7)	–

Values are presented as mean (standard deviation). BACS = Brief Assessment of Cognition in Schizophrenia; BNSS = Brief Negative Symptom Scale; CDS = Calgary Depression Scale for Schizophrenia; HC = healthy control subjects; PANSS = Positive and Negative Syndrome Scale; PSP = Personal and Social Performance scale; SHRS = St-Hans Rating Scale; SNS = Self-evaluation of Negative Symptoms; SZ = patients with schizophrenia.

**Table 2:** Demographical and clinical characteristics of patients and control subjects in Session 2

	Session 2			
	Berlin		Geneva	
	SZ (*n* = 28)	HC (*n* = 26)	SZ (*n* = 39)	HC (*n* = 29)
Demographics				
Age, years	38 (10.3)	37.7 (7.6)	38.6 (9.9)	38.8 (7.4)
Sex, % male	80	70	70	80
Education, years	13.04 (3.1)	14.8 (2.6)	12.6 (2.96)	14.8 (1.6)
Illness duration, years	13.7 (10.08)	–	13.6 (8.03)	–
Clinical variables				
BNSS				
Apathy	16.9 (10.2)	1.8 (2.5)	12 (8.1)	0.5 (1.02)
Diminished expression	9.1 (6.9)	0.8 (1.6)	7.2 (6.4)	0.8 (1.8)
PANSS				
Negative	18.5 (7.5)	8.2 (1.7)	15.5 (5.7)	7.4 (0.8)
Positive	12.04 (4.8)	7.4 (0.8)	9.9 (2.98)	7.1 (0.3)
Total	57.3 (17.8)	32.7 (3.2)	45.6 (9.6)	30.7 (1.3)
SNS				
Apathy	7.8 (4.4)	2 (1.9)	7.6 (4.9)	2.4 (2.03)
Diminished expression	7.04 (4.2)	2.6 (2.4)	6.8 (3.6)	2.2 (2.2)
Total	14.8 (8.1)	4.6 (3.8)	14.3 (7.9)	4.6 (3.5)
CDS, total	3.2 (3.7)	0.9 (1.3)	2.1 (2.6)	0.2 (0.7)
BACS, total, *Z*-score	−0.9 (1.4)	0.7 (1.3)	−1.6 (1.1)	0.9 (1.2)
PSP, total	57.04 (19.7)	86.1 (9.8)	57.2 (14.1)	92.8 (3.8)
SHRS, parkinsonism score	2.1 (4.4)	–	3.95 (4.9)	–
Risperidone equivalent	5.2 (3.8)	–	4.8 (2.6)	–

Values are presented as mean (standard deviation). BACS = Brief Assessment of Cognition in Schizophrenia; BNSS = Brief Negative Symptom Scale; CDS = Calgary Depression Scale for Schizophrenia; HC = healthy control subjects; PANSS = Positive and Negative Syndrome Scale; PSP = Personal and Social Performance scale; SHRS = St-Hans Rating Scale; SNS = Self-evaluation of Negative Symptoms; SZ = patients with schizophrenia.

For Session 2, patients in Berlin had higher scores on apathy, as measured by the BNSS, than patients in Geneva (*W* = 713, *P* = 0.03), in addition to a higher total BNSS score (*W* = 704.5, *P* = 0.04). Patients in Berlin also had trend-level higher positive symptoms as measured by the PANSS (*W* = 695.5, *P* = 0.056), in addition to a higher PANSS total score (*W* = 749.5, *P* = 0.0098). Patients in Geneva again had higher parkinsonism (*W* = 306.5, *P* = 0.002) and lower cognition (BACS; *W* = 711, *P* = 0.036).

### Behavioural results

To assess reaction times in Session 1, we performed an ANOVA on a mixed effects model, with group, study site and reward condition (no reward, small reward or large reward) as fixed effects and with participant as a random effect. We found a significant main effect of condition [*F*(296,2) = 171.3, *P* < 0.0001], with all participants being faster as predicted reward magnitude increased (Supplementary Fig. 3). There was also a main effect of group [*F*(148,1) = 25.9, *P* < 0.0001], with patients being slower than control subjects. A three-way interaction [*F*(296,2) = 4.47, *P* = 0.01] showed that control participants in Geneva were faster than control subjects in Berlin in all three conditions (all *P* < 0.024). Moreover, patients in Geneva were faster than those in Berlin only in the no-reward condition (*P* = 0.0008).

Next, we compared reaction times between sessions and found a significant three-way interaction between group, session and study site [*F*(590,1) = 8.43, *P* = 0.0038]. Although all participants were generally faster in Session 2, this increase in speed was significant only for patients in Berlin (*P* < 0.0001).

For accuracy in Session 1, there was a main effect of group [*F*(452,1) = 6.04, *P* = 0.01], with patients being slightly less accurate than control subjects [M_Acc HC_ = 0.92 (SD = 0.09), M_Acc SZ_ = 0.89 (SD = 0.13)]. In the comparison between sessions, there were no significant effects [patients became slightly more accurate and control subjects slightly less accurate in Session 2: M_Acc HC_ = 0.91 (SD = 0.28), M_Acc SZ_ = 0.907 (SD = 0.29)]. Finally, in Session 1, patients won slightly less in total than control subjects [*F*(148,1) = 6.42, *P* = 0.01, M_total HC_ = 39.9 CHF (SD = 3.9), M_total SZ_ = 37.8 CHF (SD = 5.4)]. This effect was also observed when we compared between sessions: patients won less than control subjects [*F*(118,1) = 6.93, *P* = 0.0096].

### Functional MRI results

#### Adaptive coding of reward

Supplementary Table 3 summarizes the voxel-wise whole-brain one-tailed *t*-test across all participants for the adaptive coding contrast (pmod small reward − pmod large reward) in Session 1. Similar to our previous work, we observed adaptive coding in the striatum and visual areas (Supplementary Fig. 4 shows the overlap between the present sample and the one used for the ROI mask).

#### Session 1: patients with schizophrenia show reduced adaptive coding in the striatum

As described above, we used a mask from an independent sample to define the ROIs. We extracted the activations within these three ROIs using the Marsbar toolbox and compared them using ANOVAs on linear regression models, with study site and group as factors. See the Supplementary material for any effects concerning study site.

Using boxplots for each of the four conditions (two groups and two study sites), we identified nine outlier participants, who were not included in this analysis: three patients and four control subjects in Berlin, and one patient and one control subject in Geneva. Outliers were defined as values 1.5 × interquartile range below the first quartile or above the third quartile.

**Figure 2:**
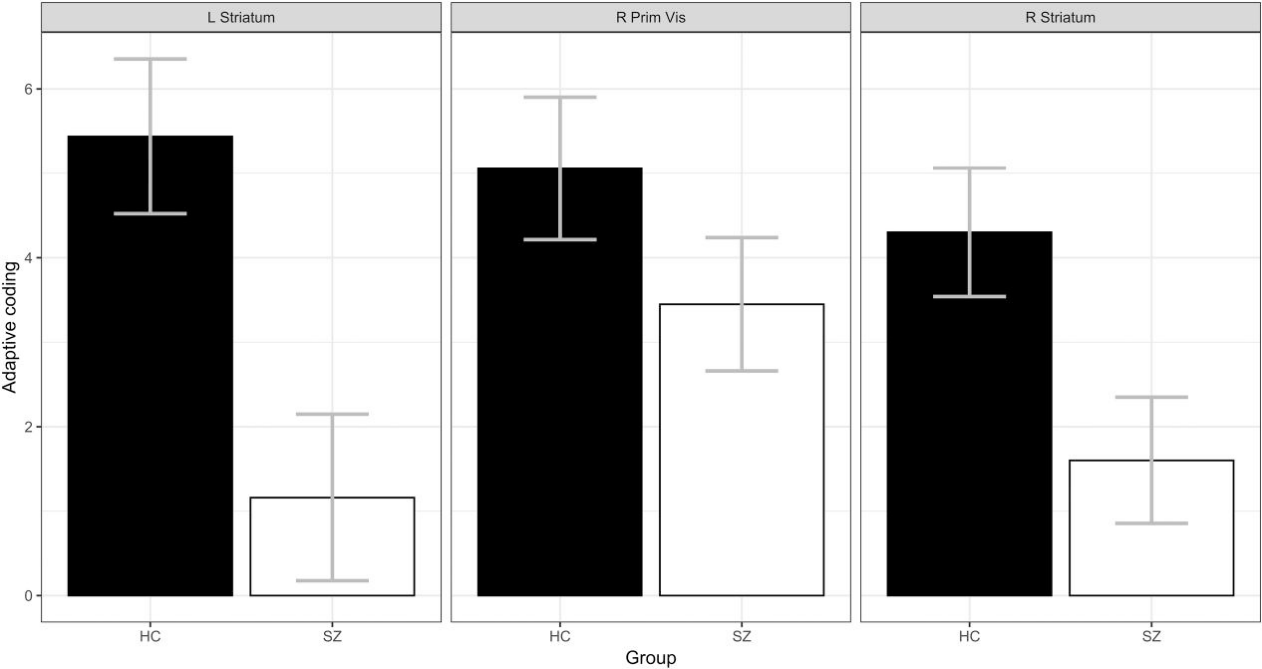
**Adaptive coding.** Session 1 (full sample). Adaptive coding (contrast: pmod narrow range − pmod wide range) in the left and right striatum and the right occipital cortex in patients with schizophrenia (SZ) and healthy control subjects (HC). Higher values correspond to a bigger difference between the blood oxygenation level-dependent (BOLD) slopes in the narrow and wide reward ranges. Error bars represent the standard error.

In the right striatum (Fig. 2), we found a main effect of group [*F*(1,139) = 5.9, *P* = 0.017], with patients showing weaker adaptive coding than control subjects. No other effects were significant (both *P* > 0.8). Likewise, in the left striatum (Fig. 2), we observed a main effect of group [*F*(1,139) = 9.29, *P* = 0.0027], again with patients showing weaker adaptive coding than control subjects. No other effects were significant (both *P* > 0.65). Conversely, no effect of group was found in the right primary visual cortex [Fig. 2; *F*(1,139) = 1.68, *P* = 0.2 (nor any other effects, both *P* > 0.5)].

Thus, this analysis replicates our previous findings of reduced adaptive coding of reward in striatal regions of patients with schizophrenia and shows that in visual areas that also encode reward adaptively, adaptation is preserved in patients.

#### Patients with schizophrenia are specifically impaired in the narrow-range context

Following the results of our previous work, we explored whether reduced adaptive coding of reward in patients was attributable to abnormal responses in the narrow range, in the wide range, or both. We thus compared the slope of brain activity increase elicited by low rewards with that elicited by high rewards using an ANOVA on a mixed effects model, with group, study site and reward range (pmod low or pmod high) as fixed effects and with participant as a random effect. For the ROIs, we used only the two striatal regions, where patients differed from control subjects. We excluded the same nine participants as in the analysis above (when outliers are removed based on the box plot procedure, similar results are obtained).

**Figure 3:**
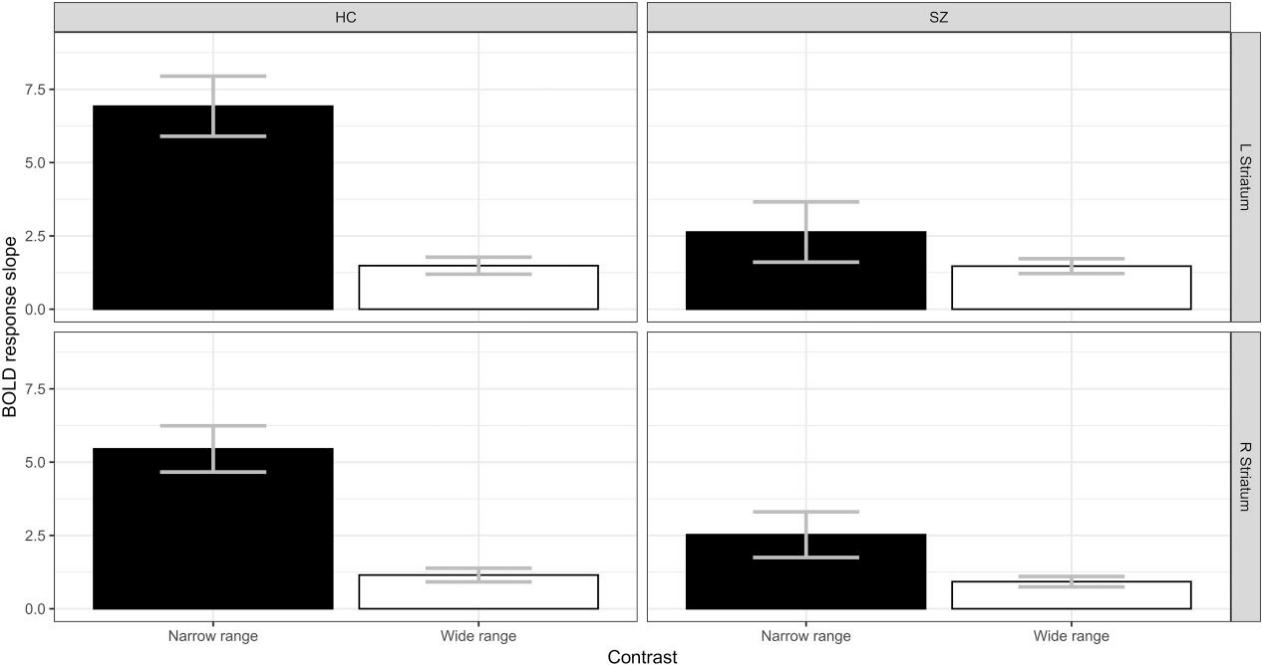
**Range adaptation.** Session 1 (full sample). Reward response blood oxygenation level-dependent (BOLD) slopes in the narrow and wide reward ranges plotted separately in the left and right striatum. Patients with schizophrenia (SZ, *right*) show a shallower slope than healthy control subjects (HC, *left*) in the narrow range, but similar slopes in the wide reward range. Adaptive coding is preserved to some extent in patients with schizophrenia. Error bars represent the standard error.

In the right striatum (Fig. 3; also see Supplementary Fig. 5 for individual results), there was a main effect of group [*F*(1,139) = 5.8, *P* = 0.016], with patients showing shallower slopes than control subjects. There was also a main effect of reward range [*F*(1,139) = 28.6, *P* < 0.0001], with steeper slopes in the narrow range than in the wide range. Importantly, the Group × Reward range interaction was significant [*F*(1,139) = 5.9, *P* = 0.016], in line with the notion that the slope difference was larger for control subjects than for patients. The difference between the wide- and narrow-range slopes was significant in both control subjects (*P* < 0.0001) and patients (*P* = 0.029). However, the narrow-range slope was less steep in patients than in control subjects (*P* = 0.0007), with no difference between groups for the wide-range slopes (*P* = 0.8).

Similar results were observed for the left striatum (Fig. 3). Again, we observed a main effect of group [*F*(1,139) = 6.4, *P* = 0.01] and reward range [*F*(1.139) = 21.6, *P* < 0.0001] and an interaction between the two [*F*(1,139) = 9.3, *P* = 0.0027]. Here, we observed adaptive coding only in control subjects (*P* < 0.0001), but not in patients (*P* = 0.2). This was because patients had significantly shallower slopes in the narrow range than control subjects (*P* = 0.0001), with no difference in the wide range (*P* = 0.99). Together, these findings corroborate our previous work by showing that adaptive coding deficits in patients are driven by a shallower slope in the narrow reward-range condition.

#### Retest after 3 months: improved adaptive coding in patients with schizophrenia?

Participants performed the monetary incentive delay task a second time ∼3 months after the first. One hundred and twenty-two participants performed the monetary incentive delay in both sessions: in Germany, 28 patients and 26 controls; and in Switzerland, 39 patients and 29 controls. Thus, 30 participants dropped out between the two sessions: 11 controls (nine in Berlin and two in Geneva) and 19 patients (eight in Berlin and 11 in Geneva).

We compared the slopes of the BOLD response in the narrow and wide reward ranges between sessions, using ANOVAs on mixed-effect models, similar to the second analysis above, for the right and left striatum, with group, study site, reward range and session as fixed effects and with participants as a random effect.

Box plots for the eight conditions (two groups, two study sites, two reward ranges and two sessions) identified 14 outlier participants, who were excluded from the analysis: three control subjects and four patients in Berlin, and one control subject and six patients in Geneva (Supplementary Fig. 6).

**Figure 4:**
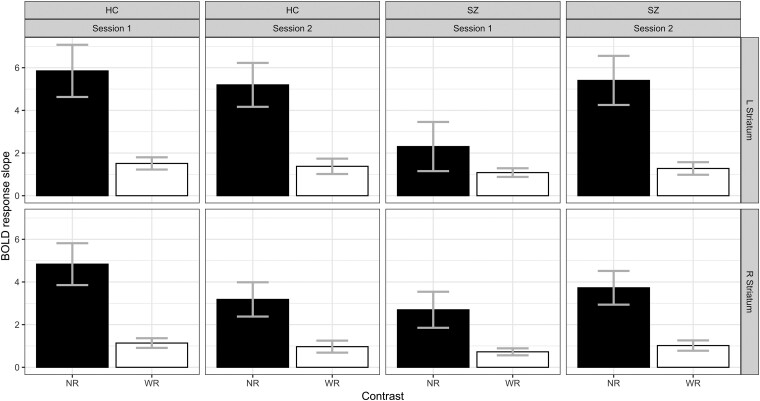
**Comparing sessions 1 and 2.** Blood oxygenation level-dependent (BOLD) response slopes in the narrow (NR) and wide (WR) reward ranges separately, in the left and right striatum. Error bars represent the standard error.

In the right striatum (Fig. 4), we found a main effect of reward range, in addition to a Group × Session interaction [*F*(1,312) = 4.2, *P* = 0.041]. Closer inspection showed that the difference between patients and control subjects observed in Session 1 disappeared in Session 2 (*P* = 0.4).

In the left striatum, in addition to the main effect of reward range, we also observed a Group × Session interaction [*F*(1,312) = 4.2, *P* = 0.043], showing no change in slope in control subjects (*P* = 0.6), but a change in patients (*P* = 0.017), such that the difference between the groups observed at Session 1 disappeared at Session 2 (*P* = 0.66). These findings appear to suggest that patients improved their adaptive coding between sessions.

Given that comparing between sessions increased the number of comparisons and the number of outliers, we performed a separate analysis on the results of Session 2 only. Eight outliers were identified using boxplots: one patient in Berlin, and one control subject and six patients in Geneva.

Confirming the above findings, in both the right and left striatum (Supplementary Fig. 7) we found a main effect of reward range [*F*(1,110) = 20.8, *P* < 0.0001 and *F*(1,110) = 28.2, *P* < 0.0001, respectively], but no main effects of group, or Group × Reward range interactions (all *P* > 0.3). Thus, in Session 2, adaptive coding was comparable between patients and control subjects.

#### Correlations

We found no significant correlations between the adaptive coding contrast (or the slope of the brain response to rewards received in narrow or wide ranges) on the one hand and symptoms or task performance on the other, or potentially confounding variables, such as cognition or chlorpromazine equivalents (all ρ < 0.17, all *P* > 0.13; Supplementary Table 4). The absence of correlations between slopes of brain activity and symptoms, in addition to task performance, was confirmed in the reduced sample of 122 in Session 1 (Table 3; correlations are presented for the mean value of the left and right striatal activity).

**Table 3:** Correlation matrix for Sessions 1 and 2

	Session 1						Session 2					
	Adaptive coding		Narrow range		Wide range		Adaptive coding		Narrow range		Wide range	
**Symptoms**												
BNSS total	ρ = 0.11, *P* = 0.38		ρ = 0.17, *P* = 0.17		ρ = 0.17, *P* = 0.2		ρ = 0.49, *P* < 0.00001		ρ = 0.43, *P* = 0.00036		ρ = 0.14, *P* = 0.29	
BNSS apathy	ρ = 0.061, *P* = 0.64		ρ = 0.13, *P* = 0.3		ρ = 0.15, *P* = 0.26		ρ = 0.52, *P* < 0.00001		ρ = 0.49, *P* < 0.00001		ρ = 0.2, *P* = 0.12	
BNSS diminished expression	ρ = 0.18, *P* = 0.15		ρ = 0.2, *P* = 0.11		ρ = 0.14, *P* = 0.29		ρ = 0.32, *P* = 0.0097		ρ = 0.27, *P* = 0.033		ρ = 0.031, *P* = 0.81	
PANSS total	ρ = −0.12, *P* = 0.35		ρ = −0.089, *P* = 0.48		ρ = 0.1, *P* = 0.42		ρ = 0.46, *P* = 0.00013		ρ = 0.39, *P* = 0.0013		ρ = 0.01, *P* = 0.94	
SNS total	ρ = 0.055, *P* = 0.67		ρ = 0.12, *P* = 0.34		ρ = 0.061, *P* = 0.64		ρ = 0.3, *P* = 0.018		ρ = 0.31, *P* = 0.014		ρ = 0.09, *P* = 0.48	
BACS (cognition)	ρ = 0.05, *P* = 0.7		ρ = 0.00014, *P* = 0.99		ρ = 0.06, *P* = 0.64		ρ = 0.078, *P* = 0.54		ρ = 0.065, *P* = 0.61		ρ = 0.22, *P* = 0.076	
**Task performance**												
	**SZ**	**HC**	**SZ**	**HC**	**SZ**	**HC**	**SZ**	**HC**	**SZ**	**HC**	**SZ**	**HC**
RT speeding	ρ = 0.066, *P* = 0.61	ρ = 0.085, *P* = 0.54	ρ = 0.089, *P* = 0.49	ρ = 0.069, *P* = 0.62	ρ = 0.27, *P* = 0.038	ρ = 0.22, *P* = 0.13	ρ = −0.051, *P* = 0.69	ρ = −0.0026, *P* = 0.99	ρ = −0.041, *P* = 0.75	ρ = 0.0007, *P* = 0.99	ρ = 0.28, *P* = 0.029	ρ = 0.072, *P* = 0.6

Correlations in the reduced sample of participants (*n* = 122, excluding outliers) between the extracted values for the adaptive coding contrast (pmod small reward − pmod large reward) and the extracted values for the narrow and wide reward range conditions (mean of the two striata) on the one hand, and clinical and task performance variables on the other. BACS = Brief Assessment of Cognition in Schizophrenia; BNSS = Brief Negative Symptom Scale; HC = healthy control subjects; PANSS = Positive and Negative Syndrome Scale; RT = reaction time; SNS = Self-evaluation of Negative Symptoms; SZ = patients with schizophrenia.

**Figure 5:**
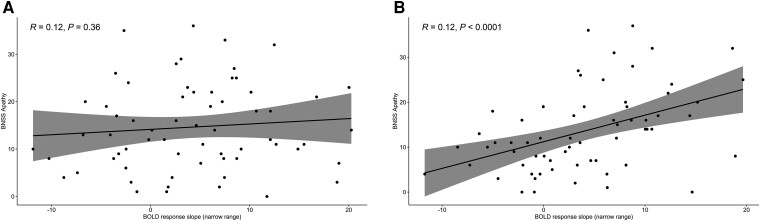
**Correlations.** Spearman correlations between the apathy dimension of negative symptoms [measured by the Brief Negative Symptom Scale (BNSS)] and the mean slope of blood oxygenation level-dependent (BOLD) activity in the left and right striatum (mean) in the narrow reward range in Session 1 (**A**) and Session 2 (**B**).

In Session 2, however, patients showed a strongly significant positive correlation between motivational negative symptoms as measured by BNSS and adaptive coding (in addition to the narrow-range slope, but not the wide-range slope) in both the left (ρ = 0.45, *P* < 0.0002) and right (ρ = 0.48, *P* < 0.0001) striatum (Fig. 5). Similar results were obtained with the PANSS negative subscale and SNS apathy subscale, in addition to total negative symptoms measured by the BNSS (Table 3). All these correlations survived correction for multiple comparisons, and the symptoms shown by patients remained stable from Session 1 to Session 2 (Tables 1 and 2).

In addition, patients in Session 2 showed positive correlations between the adaptive coding contrast (and the response slope in the narrow range) and diminished expression score (BNSS), although these correlations did not survive correction for multiple comparisons. There were no correlations with positive symptoms (PANSS) or cognition (BACS; Table 3). In sum, adaptive coding by patients, driven by the increase in slope in the narrow reward range was related to stronger negative symptoms, but not to other symptom domains.

It should be noted that similar results, in terms of group and condition differences and the correlations, were obtained when using different striatal ROIs, for example, the six ROIs specified by Mawlawi *et al*.,^29^ or the striatum from the reward-sensitive regions in the same participants (obtained when using the contrast pmod low + pmod high, as in our previous work^8,9^; see Supplementary material section ‘Supplementary results using a different set of ROIs’).

## Discussion

We investigated adaptive range coding of reward in a large sample of patients with schizophrenia (*n* = 86) and healthy controls (*n* = 66) using the monetary incentive delay task. We confirm our previous findings, demonstrating that adaptive coding is impaired in patients compared with control subjects in the striatum and that this impairment is driven by the difficulty for patients to increase their brain response to smaller rewards in the narrow reward range to the same extent as control subjects.

The present study goes beyond the previous work in multiple ways. First, adaptive coding was present (albeit reduced) in our patients, because a significant difference between narrow and wide reward range slopes was observed in the striatum. Second, adaptive coding appeared to be preserved in our patients in the right primary visual cortex, because the difference between patients and control subjects did not reach significance in this area.

### Reduced (striatum) versus preserved (visual cortex) adaptive coding in patients

Our neural results are in line with our previous and ongoing work. We have already shown reduced adaptive coding in patients with schizophrenia within striatal regions, the hub of reward processing,^8,9^ and that this reduction is driven by a shallower slope in the narrow reward range in particular.^15^ The present work confirms these findings in a large group of patients.

Whole-brain analyses of the adaptive coding contrast show activation in the ventral and dorsal striatum (the putamen in particular) (Supplementary Fig. 4B). Other neuroimaging work on reward adaptation has also localized it to the ventral part of the striatum, showing contextual sensitivity in prediction error signalling.^30-33^ Our previous work has also localized reward-adaptive coding in the dorsal striatum in the monetary incentive delay task.^8,9^ Hypoactivation of the putamen has been reported previously in schizophrenia during reward processing,^34-36^ and the dopaminergic dysfunction in schizophrenia is located mostly in the dorsal striatum.^37^ Thus, the putamen, similar to the ventral striatum (and possibly through anatomical proximity) might encode reward adaptively. Such an adaptive response would be of particular relevance for action selection. The dorsal part of the striatum has been associated with cognitive flexibility and action selection, crucial for adaptation to changing environmental demands. Thus, the ventral striatum is involved in integration of rewarding, emotional and social contextual information,^38-41^ which, interacting with the dorsal striatum and corresponding cortical areas, would promote learning, flexible decision-making and adapted goal-directed behaviour.

Apart from the striatum, we also observe adaptive coding in the right primary visual cortex. Previous work has shown the involvement of sensory areas, and early visual processing areas (V1) in particular during the processing of rewarding information.^42^ Rewarded stimuli are known to enhance attention allocation and visual processing.^43,44^ Interestingly, although our patients showed general deficits in attention processing [BACS symbol coding difference between patients and control subjects, *F*(1,118) = 56.2, *P* < 0.0001], the activity in their visual areas adapted to reward context statistically in a similar manner to healthy control subjects. Given that psychiatric prevalence in our control subjects was assessed through self-report only, it remains possible that they were unaware of affected family members or symptoms. This might lead to reduced group differences between them and the patients in our cohort. However, our supplementary analyses do show reduced adaptive coding in patients in left and right visual associative areas, visuo-motor areas and the supramarginal gyrus (involved in attentional and motor orienting^45,46^) (Supplementary Table 5 and Supplementary Fig. 8). Thus, further along the visual hierarchy patients show reduced modulation by reward context. Additionally, we also find lower adaptive coding in patients in the right anterior cingulate cortex, a region previously reported to show reward-related contextually modulated activity.^33,47^

### Adaptation in different contexts (ranges)

The reduction in adaptive coding was attributable to a shallower striatal response slope in the narrow range in patients relative to control subjects. How can we explain this effect? *Ex ante*, one might have expected the patients to show a deficit in the wide rather than the narrow reward range. More specifically, one could imagine a deficit in the precise coding of multiple rewards, which would be more of a problem for the wide range than for the narrow range (Fig. 1B). This, in turn, could be related to apathetic symptoms, because, for example, actions resulting in more reward would not be perceived as such. Interestingly, however, we observe no difference in the wide-range condition between patients and control subjects in either the present data or the data of Kirschner *et al*.^8,15^

Our protocol does not allow us to differentiate between range adaptation and mean adaptation. It is thus not entirely possible to deduce whether the shallow slope in the narrow-range condition is attributable to the mean or the range of the rewards in this condition being on average smaller than in the wide-range condition. On the one hand, it is possible that patients find it particularly difficult to represent the gratifying nature of small rewards. Thus, small-reward contexts would not produce a similar increase in brain activity to large-reward contexts. This might relate to the apathetic symptoms of patients, in that many everyday activities (shopping, cleaning and personal hygiene) involve modest rewards; it is possible that the inability to represent the rewarding nature of such activities accurately results in a lack of motivation for them. Our data, however, do not appear to support this hypothesis, because no correlation was observed between apathy and reduced adaptation in the narrow range in Session 1.

On the other hand, patients could have a selective deficit in adapting their brain response to a narrow range, irrespective of the reward magnitude. Previous work has claimed that range adaptation depends on a balanced excitation–inhibition ratio^10,48,49^ and that this balance appears to be disturbed in schizophrenia.^50-53^ This excitation–inhibition imbalance, together with processing-speed deficits characteristic of schizophrenia,^54^ could result in a deficit of contextual range adaptation in the narrow range specifically where a more abrupt up- or downregulation of brain activity is required.

### Changes between sessions

One interesting question we hoped to answer with our study is whether adaptive coding remains stable over time. In healthy control subjects, we observed either no change in adaptive coding between sessions or a slight reduction in the narrow-range slopes (Fig. 4 and Supplementary Fig. 6) This change might be attributable to habituation,^55-57^ whereby a reduced striatal response is observed upon repeated receipt of reward. Thus, habituation would primarily affect small rewards, resulting in a shallower slope in the narrow range, and consequently, reduced adaptive coding.

Interestingly, such habituation was not observed in patients. Instead, we observed an ‘improvement’ in patients, whose slopes in the narrow reward range became steeper than in Session 1 (showing robust adaptive coding). Thus, patients appear to benefit from retesting to scale their brain responses more sensitively to available rewards. Previous work in different domains has also documented improved brain response in patients with schizophrenia upon retesting.^58-60^ This is, to our knowledge, the first longitudinal study of reward adaptation. We thus show changes in opposite directions in healthy participants and in patients with schizophrenia.

The finding of an improvement in adaptive coding might suggest that this deficit is amenable to treatment and thus open opportunities for rehabilitation targeting improvements in reward processing and motivation.

### Relationship to symptoms

In Session 1, we found no correlations between adaptive coding and symptoms or task performance by patients. In Session 2, in contrast, we found a strong correlation between the slope in the narrow reward range and negative symptoms, assessed by two specialized measures (BNSS and PANSS) and self-assessed (SNS), and a modest correlation with global functioning, measured with the PSP scale and the GAF scale. Thus, patients with steeper slopes had stronger symptoms and worse functioning. This paradoxical finding might be explained by the fact that across the two sessions patients with stronger negative symptoms had steeper slopes in the narrow reward range and, on average, in Session 2, those with stronger symptoms slightly increased their slopes, whereas those with weaker symptoms slightly decreased their slopes. It thus appears that repeated testing might enhance individual differences between participants, leading to either overadaptation in those with an initially stronger response or reduced adaptation in those with an initially weaker response.

Why would a steeper slope in the narrow range be associated with increased symptoms? Excessively steep slopes in the narrow range are also maladaptive, because they lead to an incomplete representation of higher reward values, for which precision is lost (increasing amounts of reward will not lead to an increased signal because the response is already at ceiling; Fig. 1B). Some of our patients demonstrated steeper slopes than control subjects in Session 2 (Supplementary Fig. 6). Wang *et al*.^10^ also report increased behavioural adaptive coding of reward pleasantness judgements in patients with chronic schizophrenia compared with healthy control subjects (interestingly, in first-episode patients adaptive coding was reduced relative to control subjects). A visual inspection of their results (Figure 2b and c in Wang *et al*.^10^) appears to confirm an increase in slope in the narrow range in patients compared with control subjects, with no difference in the wide range between groups.

Thus, one explanation of the association between symptoms and adaptive coding is that patients with higher apathetic symptoms have apparently lost the capacity to discriminate precisely between the largest rewards within the narrow range.

### Limitations

Although the results of this study demonstrated statistical significance, caution should be exercised when generalizing the results that approached the conventional significance threshold. A task distinguishing between mean and range adaptation would provide more precise insights. Nevertheless, our results confirm and extend our previous findings, showing that differences between patients and control subjects stem from perturbed narrow-range adaptation.

## Conclusion

In conclusion, our results confirm reduced adaptive coding in striatal regions in patients with schizophrenia. Importantly, we show that patients appear to benefit from retesting, improving their adaptation. However, this change in brain response might be maladaptive in patients, reflecting faster saturation and an inability to represent the full reward range adequately, rather than improved adaptation.

## Supplementary Material

awae112_Supplementary_Data

## Data Availability

Data are available from the corresponding author upon reasonable request.
