# Supplementary material for: Adaptive coding of reward in schizophrenia, its change over time and relationship to apathy
Source: Brain. 2024 Apr 12;147(7):2459–70. doi: 10.1093/brain/awae112 (PMC11224610; doi:10.1093/brain/awae112)
Supplement: awae112_Supplementary_Data [file awae112_supplementary_data.pdf]

The SYMONE Cohort

The analyses presented here were performed on the SYMONE cohort. The SYMONE cohort is a clinical cohort including patients with schizophrenia and healthy controls. The SYMONE cohort was designed to define behavioral and neural markers of apathy in schizophrenia. The definition of such markers depends on three main points: 1) a robust association between markers and apathy that has 2) longitudinal stability and 3) stability across centers. The design of the study was also done to test the predictive power of these biomarkers on apathy levels after nine months. The SYMONE study took place over three sessions (Session 1, Session 2 after three months and Session 3 after six months) in two centers (Berlin, Germany and Geneva, Switzerland). The study comprised a full demographical and clinical evaluation, two behavioral tasks and two functional magnetic resonance imaging (fMRI) tasks (Supplementary Table 1). Sessions 1 and 2 were composed of all three parts, while Session 3 comprised solely the demographical and clinical evaluation. The study was approved by the local ethics committees in both centers.

Patients with schizophrenia were recruited from outpatient units at the University Hospital in Geneva, Switzerland and at the Charité Hospital in Berlin, Germany. Inclusion criteria included a diagnosis of schizophrenia, clinical stability, and no hospitalization or medication change in the last four months. Major causes for secondary negative symptoms were excluded (i.e. current major depressive episode, florid psychotic symptoms and extra-pyramidal side-effects). Healthy control participants were recruited from the general population and were matched with the patient group based on their age, gender and personal and parental education. Healthy controls with a history of psychiatric or neurologic disorders were excluded. Power analyses indicated that a sample of 66 patients with schizophrenia and 32 healthy controls (with an estimated drop-out rate of 30% due to the longitudinal design of the study) per center was necessary. Due to the Covid19 pandemic, we failed to recruit that many participants. At the end of the recruitment phase, we had included 60 patients and 32 healthy controls in Geneva and 47 patients and 36 healthy controls in Berlin. Note that participant numbers in articles based on the SYMONE cohort may vary, as not all participants performed every task in every session (see Supplementary Figure 1 for the participants inclusion in this study).

Supplementary Table 1. Summary of the Data Acquired for Participants in the SYMONE Cohort

**Demographics** (including age, gender, education of participant and parents, onset and duration of illness, medication)	
**Clinical Evaluation**	
Major psychiatric disorders evaluation	Mini-International Neuropsychiatric Interview (MINI; Sheehan et al., 1998)
Negative symptoms (hetero-evaluation)	Brief Negative Symptom Scale (BNSS; Kirkpatrick et al., 2011; Strauss et al., 2012)
Positive and negative symptoms (hetero-evaluation)	Positive and Negative Syndrome Scale (PANSS; Kay, Fiszbein, & Opler, 1987)
Negative symptoms (self-evaluation)	Self-evaluation of Negative Symptoms (SNS; Dollfus et al., 2016)
Apathy	Marins Apathy Scale for clinicians (AES; Marins, 1991)
Motivation	Demotivational Beliefs Inventory (DBI; Pillny et al., 2018)
Depressive symptoms	Calgary Depression Scale (CDS; Addington, Addington, & Maticka-tyndale, 1993)
Cognition score	Brief Assessment of Cognition in Schizophrenia (BACS; Keefe, 2004)
Reaction to ambiguous situations	Intolerance of Uncertainty Scale (IUS; Freestone, 1994)
Parkinsonism symptoms	St. Hans Rating Scale (SHRS; Gerlach et al., 1993)
Global functioning	Personal and Social Performance scale (PSP; Morosini, 2000)
	Global Assessment of Functioning scale (GAF; Frances, 1994)
**Behavioral Tasks**	
Physical effort-based decision making task	Effort-based Decision Making task (Hartmann et al., 2015)
Reinforcement learning	One-Step task (similar to Frank et al., 2003)
**fMRI Tasks**	
Reward anticipation	Monetary Incentive Delay task (MID; Knutson, 2000)
Reinforcement learning	Volatile Reversal task (similar to Boehme et al., 2015)

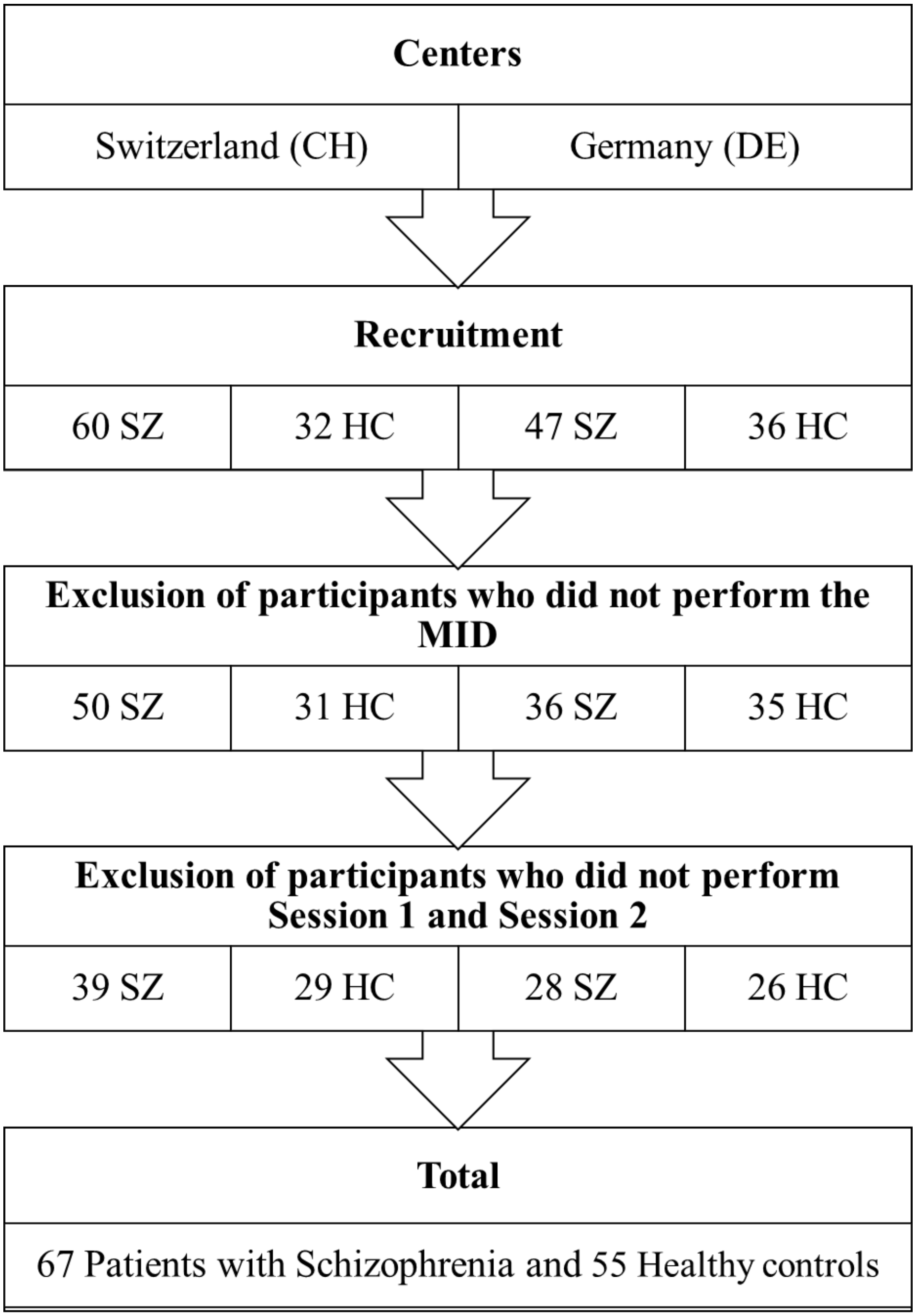

Supplementary Figure 1. Exclusion procedure of participants from the SYMONE cohort for the reward anticipation analyses presented in this paper.

Supplementary results

These results complement the fMRI results reported in the main text in the section "Retest after three months: Improved adaptive coding in patients with schizophrenia?".

In the right striatum (Figure 4), we found a main effect of Reward range, as well as a Group*Session interaction (F(1,312)=4.2, p=0.041). Closer inspection showed that the difference between patients and controls observed at Session 1 disappeared at Session 2 (p=0.4). There was also a Study site*Group interaction (F(1,104=5.9, p=0.017), showing no difference between patients and controls in Berlin (p=0.2), but a difference in Geneva (p=0.02).

In the left striatum, in addition to the main effect of Reward range, we also observed a Group*Session interaction (F(1,312)=4.2, p=0.043), showing no change in slope in controls (p=0.6), but a change in patients (p=0.017), such that the difference between the groups observed at Session 1, disappeared at Session 2 (p=0.66). These findings appear to suggest that patients improved their adaptive coding between sessions.

Since comparing between sessions increased the number of comparisons as well as the number of outliers, we performed a separate analysis only on the results of Session 2. Eight outliers were identified using boxplots: 1 patient in Berlin, 1 control and 6 patients in Geneva.

In the right striatum (Supplementary fig. 7) we found a main effect of Reward range (F(1,110)=20.8, p<0.0001) a Study site*Group interaction (F(1,110)=9.01, p=0.003), and a 3-way Study site*Group*Range interaction (F(1,110)=6.38, p=0.01), showing that adaptive coding did not remain significant for controls in Berlin (difference between slopes in the two reward ranges p=0.4), but remained significant for controls in Geneva (p=0.002). For patients, we observed strong adaptive coding in Berlin (p=0.0002), but not in Geneva (p=0.2). In both Berlin and Geneva patients had significantly steeper slopes in the narrow range condition than controls (p=0.0007 and p=0.04, respectively). No differences involving the wide range condition were found.

In the left striatum we observed the same pattern. There was a main effect of Reward range (F(1,110)=28.2, p<0.0001), a Study site*Group interaction (F(1,110)=7.9, p=0.0059) and the 3-way Study site*Group*Range interaction (F(1,110)=4.55, p=0.035. Here adaptive coding was present in controls (Berlin p=0.028; Geneva p=0.01), and in patients in Berlin (p<0.0001) but not Geneva (p=0.35). Berlin patients showed a significantly steeper slope in the narrow range than

Berlin controls (p=0.0035), whereas Geneva patients showed a significantly shallower slope than Geneva controls (p=0.049). No differences involving the wide range condition were found.

As can be seen in our Supplementary figs. 5 and 6, the two reward ranges showed different levels of variability: strong variability can be seen in the narrow range while participants are quite homogeneous in their BOLD response slopes in the wide range. We indeed observe similar variability in our previous work[8,9]. This variability is not driven by the number of different levels (i.e. the average number of different reward amounts obtained by participants per trial) present in the two ranges: ~17.1 for the narrow range and ~16.1 for the wide range. Potentially, the wide range, with its higher rewards, might be more like a baseline and possibly reflect more passive and absolute coding of reward magnitude. In contrast, the narrow range is more situation-based and can elicit various degrees of active adjustment in relation to the context. Accordingly, the narrow range context might present more variability as it depends on the interpretation of the context both across time points and across people (the subjective value of smaller rewards might show higher variability).

Supplementary results using a different set of ROIs

As a proof of concept, we conducted two other analyses based on different sets of ROIs. For the first analysis, we applied the strategy of our past work and used ROIs derived from the reward sensitive regions (obtained from the contrast [pmod small reward + pmod large reward], at a cluster defining threshold of p<0.0001). We obtained 14 regions (Supplementary Table 5). The analysis was run on Session 1 data and we used the adaptive coding contrast as the dependent variable. The boxplot procedure identified 29 outliers. An ANOVA on a mixed effects model with Group, Study site and ROI as fixed effects and Participant as random effects showed a main effect of ROI ($F(13,1547)=1.9$, $p=0.02$ and a main effect of Group ($F(1,119)=4.9$, $p=0.029$), with patients showing lower adaptive coding than controls (Supplementary Figure 8). This analysis shows reduced adaptive coding in schizophrenia in reward sensitive regions across the brain.

For the second analysis, we adopted a more restricted approach and took six striatal regions of interest (ROI) defined in Mawlawi et al., 2001: the left and right ventral striatum (lVS and rVS); left and right dorsal striatum (lDS and rDS) and the left and right posterior putamen (lpPut and rpPut). The analysis was run on Session 1 data and used the slope in the narrow and wide ranges as the dependent variables. An ANOVA on a mixed effects model with Group, Study site, Reward

range and ROI as fixed effects and Participant as random effects was used (Supplementary figure 9). It showed, as expected, a main effect of Reward range ($F(1,1628)=66.1$, $p<0.0001$), with steeper slopes in the narrow range. There was also a main effect of ROI ($F(5,1628)=3.6$, $p=0.003$), with significantly steeper slopes in the rVS with respect to both the right ($p=0.027$) and the left ($p=0.025$) posterior putamen. The difference between the lVS and the right ($p=0.075$) and left ($p=0.069$) putamen did not reach significance. No other ROI effects reached significance (all $p>0.2$). Importantly, and similarly to the analysis reported in the main text, there was also a Group * Reward range interaction ($F(1,1628)=8.77$, $p=0.003$. It showed, first, adaptive coding in both patients and controls, both groups showing a significant difference between the slopes in the wide and narrow ranges ($p=0.0001$ and $p<0.0001$, respectively). Second, patients had less steep slopes than controls in the narrow range ($p=0.021$), with no difference in the wide range ($p=0.7$). No other effects reached significance (all $F<1.99$, all $p>0.16$).

This analysis corroborates our main finding of reduced adaptive coding of reward in schizophrenia being a result of a reduced slope in the narrow range context.

Similar to the main analysis, we find no correlations (Spearman) with BNSS Apathy and the narrow range slope at Session 1:

	Left	Right
Ventral Striatum	R=0.073, p=0.56	R=0.007, p=0.96
Dorsal Striatum	R=0.11, p=0.37	R=0.097, p=0.44
Posterior Putamen	R=0.06, p=0.63	R=0.14, p=0.26

At Session 2, however, these correlations became significant in the ventral and dorsal striatum (but not the posterior putamen.

	Left	Right
Ventral Striatum	R=26, p=0.035	R=0.24, p=0.047
Dorsal Striatum	R=0.38, p=0.0017	R=0.43, p=0.0003
Posterior Putamen	R=0.09, p=0.44	R=0.14, p=0.26

Supplementary figures

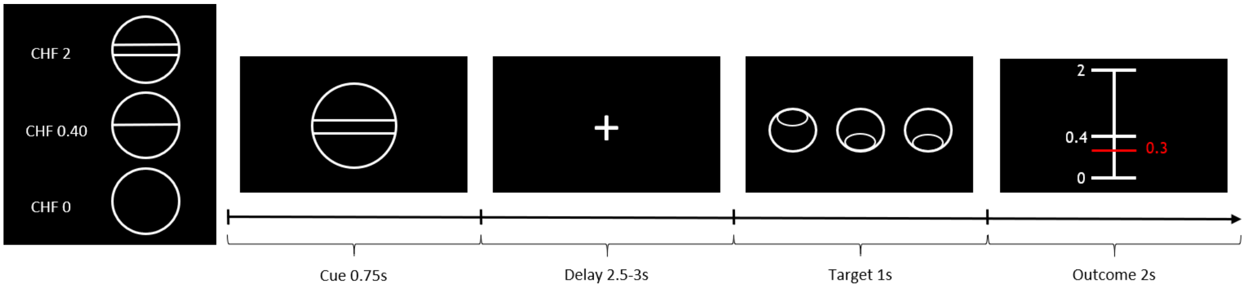

Supplementary figure 2. The Monetary Incentive Delay task (MID). The cue indicating the small reward (40¢) corresponds to the narrow reward range, and the cue indicating the large reward corresponds to the wide reward range (2CHF). To assess adaptive coding, these two outcome conditions are modelled and compared to each other.

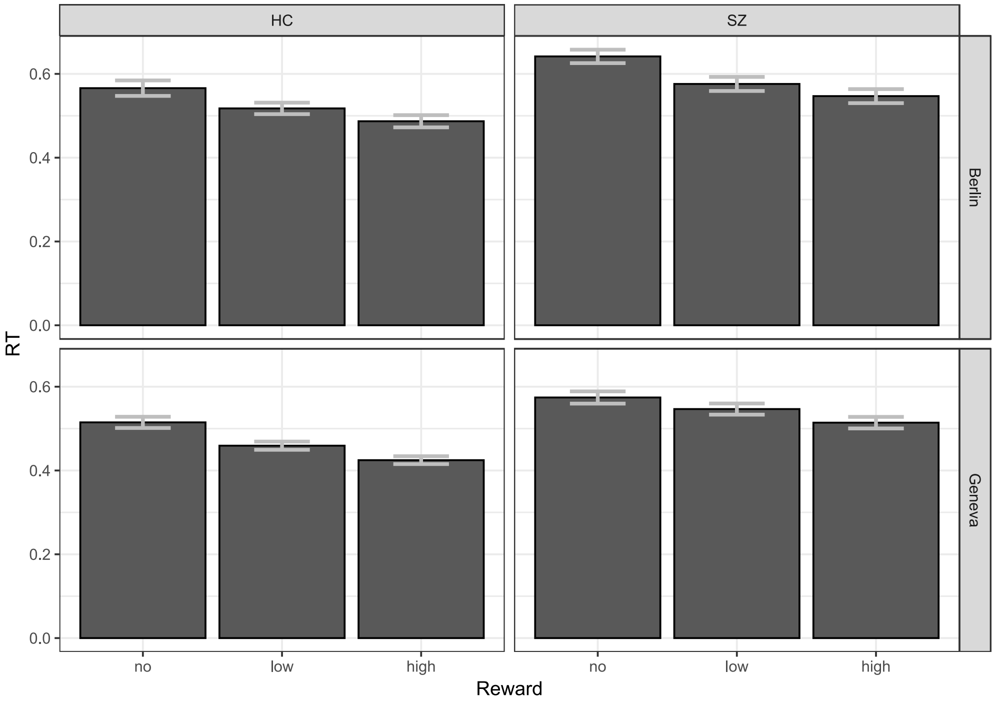

Supplementary figure 3. Reaction times. RTs in the three reward conditions (no reward, low reward, high reward), in Berlin (upper panel) and Geneva (lower panel), for healthy controls

(HC) and patients with schizophrenia (SZ). Error bars represent standard error. All participants reduced their reaction times as a function of reward condition; patients were significantly slower than controls.

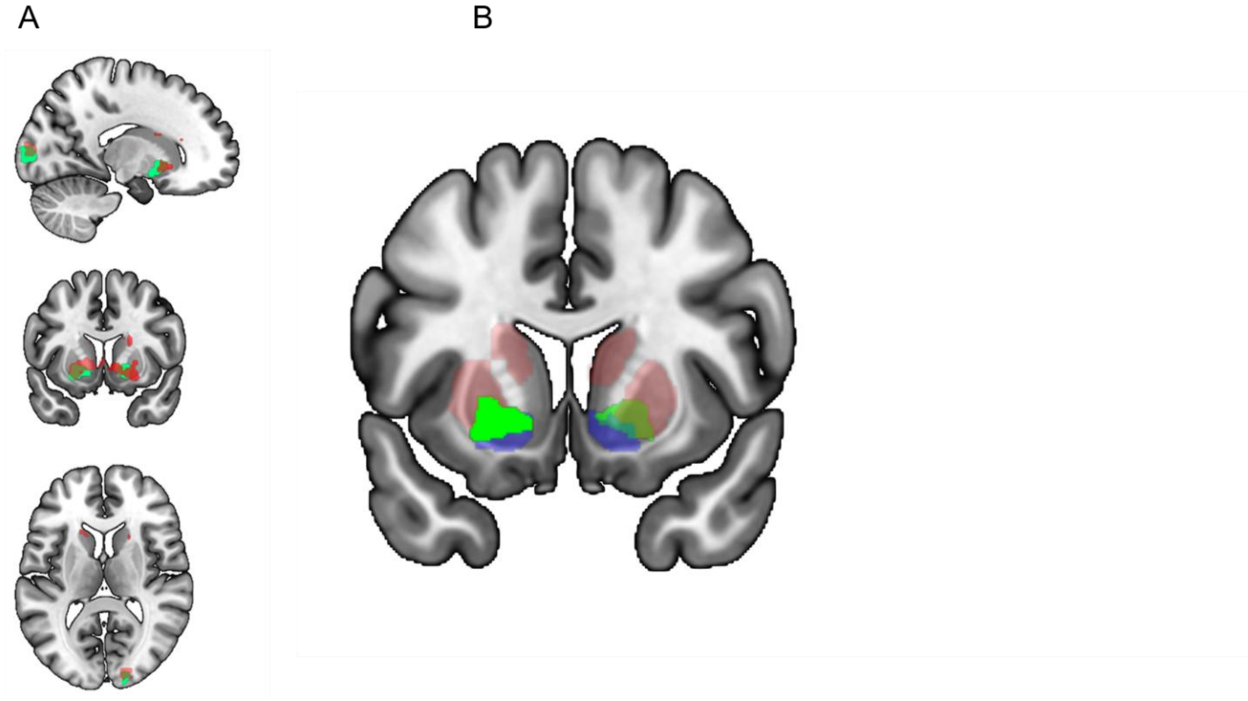

Supplementary figure 4. Regions of interest. A. Overlap between the results of the voxel-wise whole-brain analysis for the adaptive coding contrast in the present study (green) and an independent sample of healthy adults (red), in the left and right striatum and the primary visual cortex. B. Overlay of the adaptive coding activation in the present sample (green) onto the regions of the ventral (blue) and dorsal (red) striatum from Mawlawi et al., 2001[29].

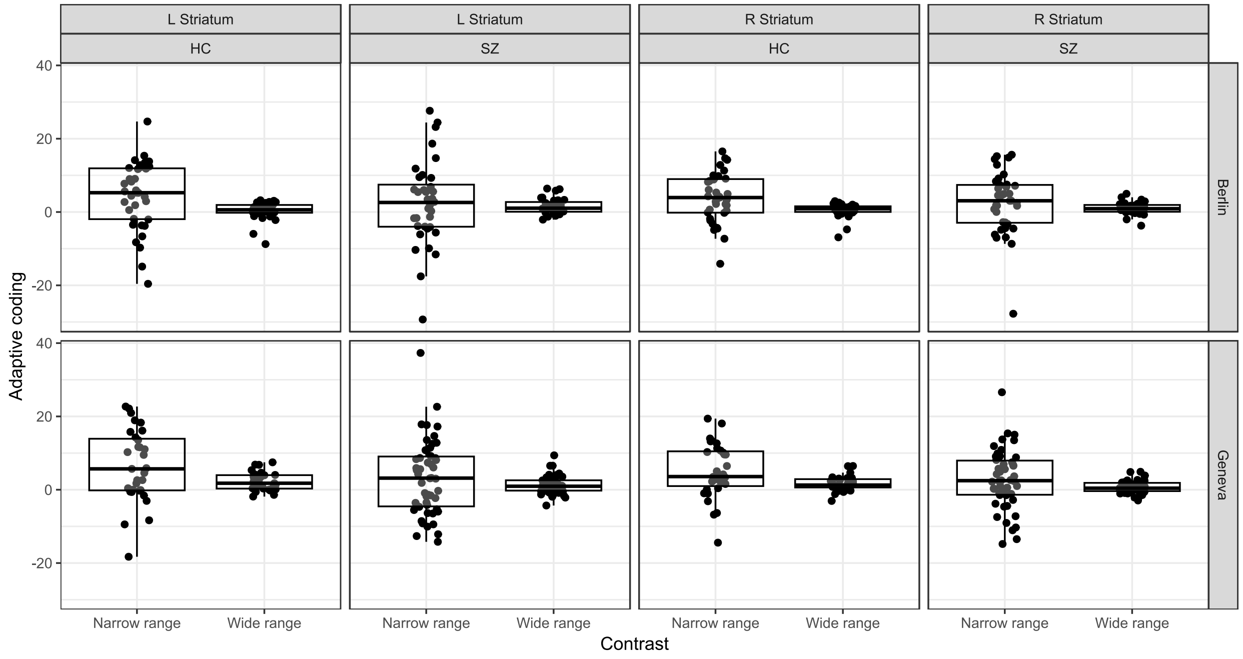

Supplementary figure 5. Session 1: Individual results. Boxplot representation of the raw data for the slopes in the narrow and wide reward ranges, separately in Geneva and Berlin, in the right and left striatum.

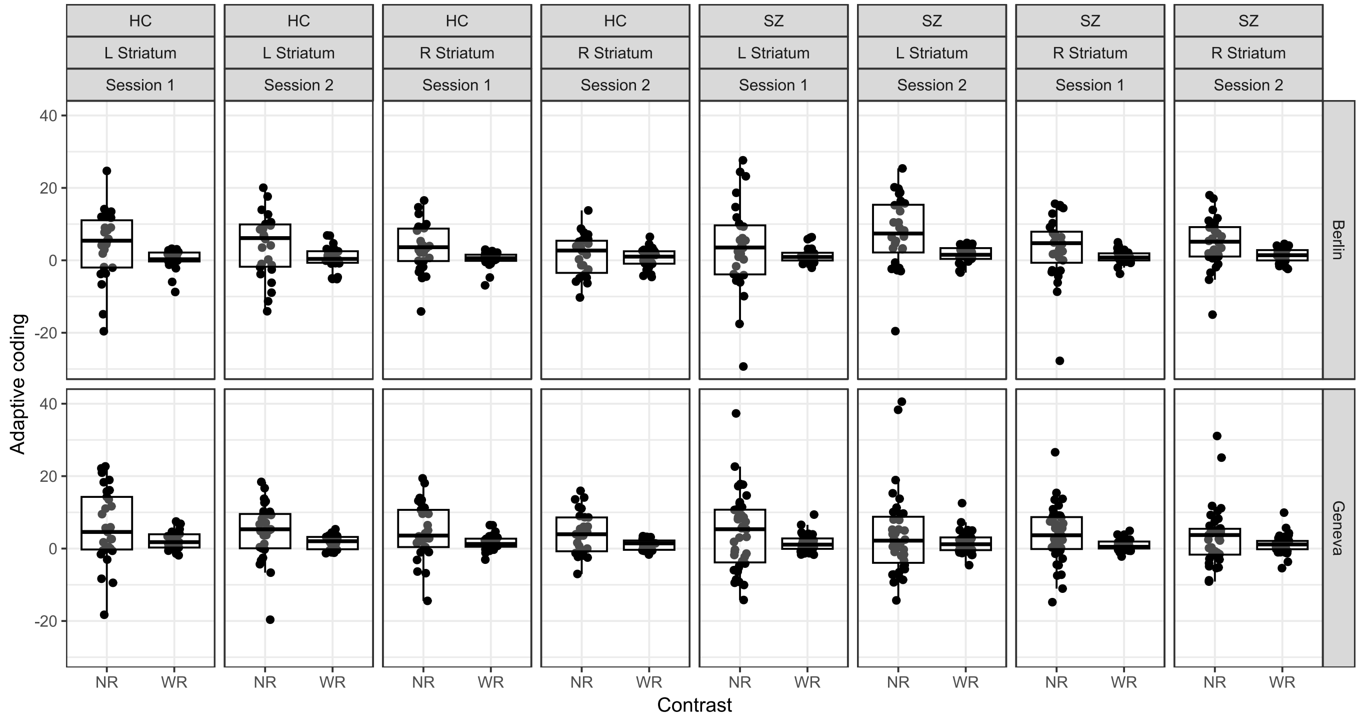

Supplementary figure 6. Comparing Session 1 and 2: Individual results. Boxplot representation of the raw data for the slopes in the narrow and wide reward ranges, separately in Geneva and Berlin, in the right and left striatum, for Session 1 and 2.

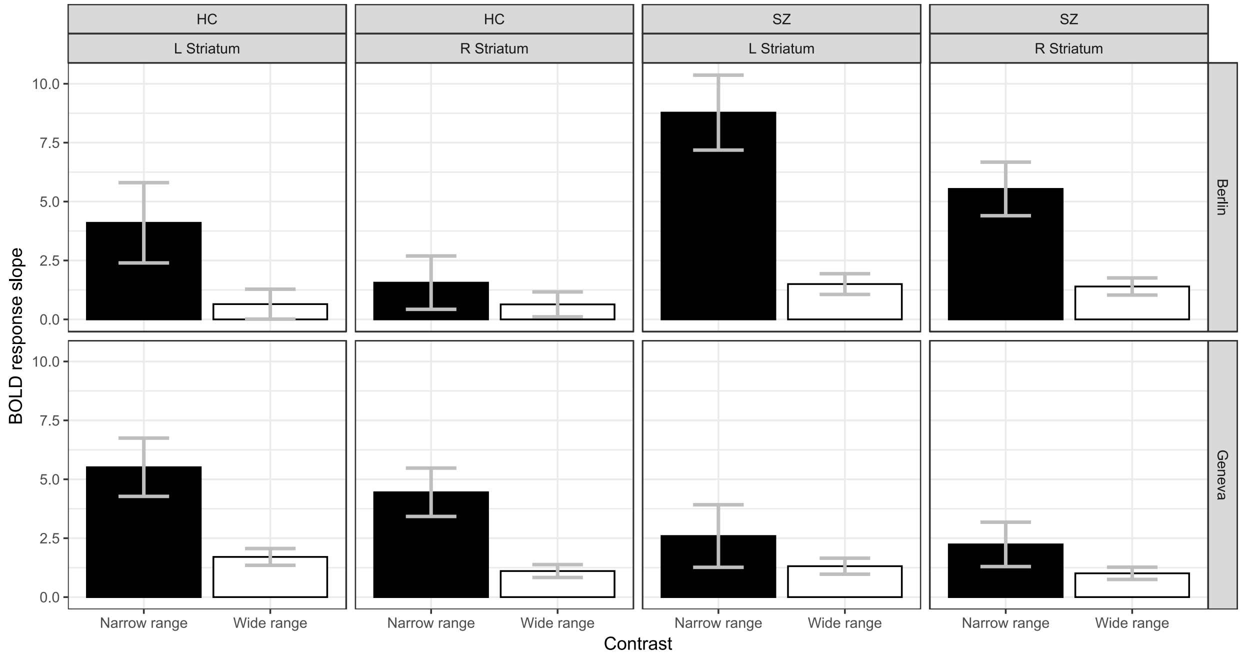

Supplementary figure 7. Comparing Session 1 and 2: Individual results. Boxplot representation of the raw data for the slopes in the narrow and wide reward ranges, separately in Geneva and Berlin, in the right and left striatum, for Session 1 and 2.

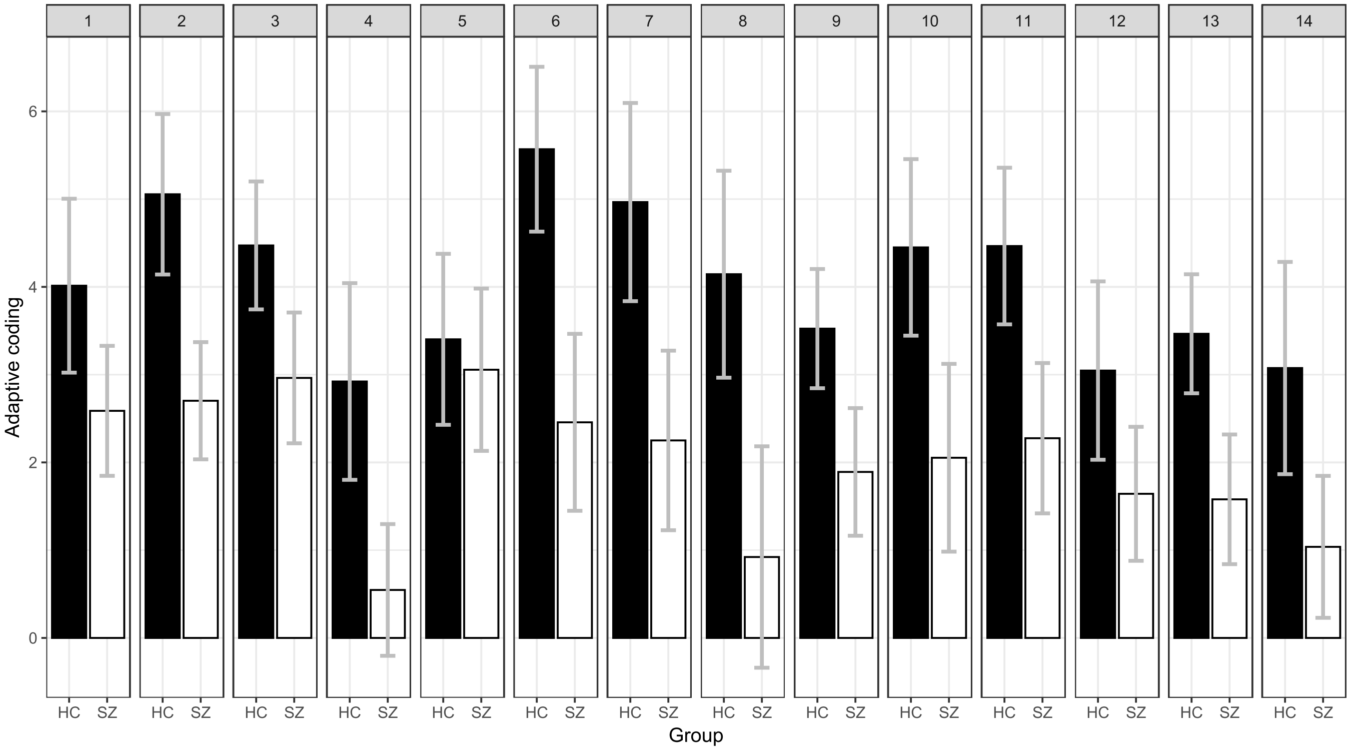

Supplementary figure 8. Session1 (full sample). Adaptive coding in patients and controls in 14 reward sensitive regions obtained from the [pmod small reward + pmod large reward] contrast (left striatum: area 6; right striatum: area 3, full set of regions cf. Suppl Table 5). Patients (in white) show reduced adaptive coding.

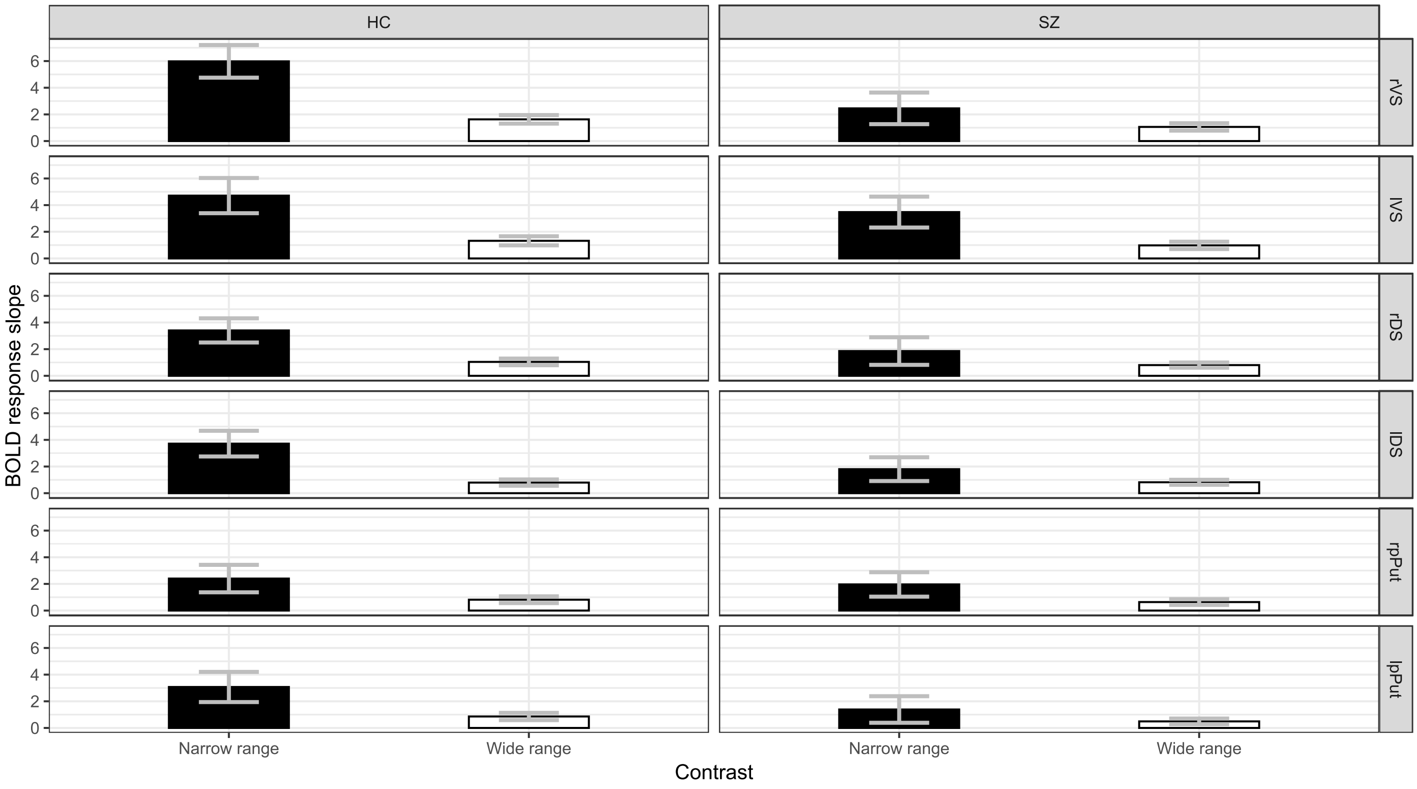

Supplementary figure 9. Session 1 (full sample). Reward response BOLD slopes in the narrow and wide reward ranges plotted separately for 6 ROIs: left and right dorsal striatum (lDS and rDS) and the left and right posterior putamen (lpPut and rpPut), in healthy controls (HC, left) and patients with schizophrenia (SZ, right). Patients with schizophrenia show a shallower slope than healthy controls in the narrow range, but similar slopes in the wide reward range.

Supplementary Tables

Supplementary Table 2. Whole brain analyses of adaptive coding across all participants in the MID task in an independent sample of 86 healthy adults

	x	y	z	Cluster size	t
Left Putamen	-18	10	-2	465	6.486685
	-22	6	-10		5.332152
	-12	16	8		4.462849
Right Caudate	10	8	-6	495	6.087996
	22	8	-8		6.011537
	20	30	14		5.656465
Right secondary visual cortex	18	-92	10	77	5.439005
	24	-92	16		3.250984

Results at $p<0.05$ FWE whole brain corrected

Supplementary Table 3. Whole brain analysis of adaptive coding across all participants (N=81)

	x	y	z	Cluster size	t
Right primary visual area	15.5	-92.5	3.5	171	6.615021
	21.5	-96.5	13.5		4.31538
Right putamen	15.5	7.5	-4.5	153	5.279498
	23.5	11.5	-8.5		3.958001
Left Putamen	-24.5	7.5	-10.5	183	4.746331
	-12.5	7.5	-6.5		4.349756

Supplementary Table 4. Correlation matrix at session 1

	Adaptive coding		Narrow range	
**Symptoms**				
BNSS total	rho=0.16, p=0.16		rho=0.17, p=0.13	
BNSS apathy	rho=0.091, p=0.42		rho=0.12, p=0.29	
BNSS diminished expression	rho=0.23, p=0.04		rho=0.21, p=0.054	
PANSS total	rho=-0.024, p=0.83		rho=0.038, p=0.74	
PANSS positive	rho=-0.16, p=0.14		rho=-0.12, p=0.3	
PANSS negative	rho=0.079, p=0.48		rho=0.098, p=0.38	
CDS (depression)	rho=0.011, p=0.92		rho=0.1, p=0.36	
SNS total	rho=0.11, p=0.32		rho=0.17, p=0.14	
SNS apathy	rho=0.073, p=0.52		rho=0.11, p=0.31	
PSP	rho=0.1, p=0.35		rho=0.056, p=0.62	
GAF	rho=0.14, p=0.21		rho=0.091, p=0.41	
BACS (cognition)	rho=0.017, p=0.88		rho=0.014, p=0.9	
**Task performance**				
	**SZ**	**HC**	**SZ**	**HC**
RT speeding	rho=-0.007, p=0.95	rho=0.089, p=0.49	rho=-0.017, p=0.88	rho=0.15, p=0.24
Accuracy	rho=0.0019, p=0.99	rho=-0.023, p=0.86	rho=0.014, p=0.9	rho=-0.083, p=0.53
Total won	rho=-0.023, p=0.84	rho=-0.058, p=0.65	rho=0.055, p=0.62	rho=-0.018, p=0,89

Supplementary Table 5. Whole brain reward-sensitive regions

		x	y	z	Cluster size	t
1	Right visual associative	25.5	-70.5	-6.5	189	7.187341
		29.5	-56.5	-8.5		5.089943
2	Right primary visual	15.5	-92.5	3.5	434	6.996754
		19.5	-94.5	15.5		6.868949
		27.5	-80.5	17.5		5.100525
3	Right Putamen	15.5	1.5	-10.5	813	6.808304
		-8.5	1.5	19.5		6.187335
		-0.5	5.5	11.5		5.850195
4	Left visual associative	-26.5	-72.5	-8.5	143	6.189017
5	Left Premotor/Supplementary Motor	-42.5	-4.5	13.5	102	6.120365
		-48.5	-0.5	3.5		4.40917
6	Left Putamen	-22.5	5.5	-10.5	394	6.096246
		-12.5	9.5	-6.5		5.691239
		-22.5	7.5	-2.5		5.459818
7	Right Premotor/Supplementary Motor	3.5	-0.5	61.5	185	5.558525
		-4.5	3.5	41.5		5.052047
		-0.5	-4.5	51.5		4.807611
8	Right Ventral Anterior Cingulate	1.5	29.5	15.5	103	5.50348
		-2.5	21.5	19.5		4.157056
		-0.5	35.5	7.5		4.126837
9	Right Visuomotor	21.5	-58.5	55.5	205	5.282889
		23.5	-52.5	61.5		4.818162
		33.5	-40.5	57.5		4.584774
10	Left Supramarginal Gyrus	-46.5	-30.5	39.5	60	5.218102
11	Right Premotor/Supplementary Motor	39.5	-4.5	55.5	319	5.116504
		37.5	-18.5	53.5		4.979479
		45.5	-28.5	45.5		4.841296
12	Left Secondary Visual	-16.5	-88.5	-6.5	39	4.630022
		-12.5	-94.5	-2.5		4.33236
13	Left Visuomotor	-18.5	-54.5	59.5	62	4.601127
		-10.5	-46.5	53.5		4.478962
14	Left visual associative	-30.5	-80.5	19.5	44	4.397856
		-20.5	-88.5	17.5		4.383892

1. Boehme R, Deserno L, Gleich T, et al. Aberrant Salience Is Related to Reduced Reinforcement Learning Signals and Elevated Dopamine Synthesis Capacity in Healthy Adults. *The Journal of Neuroscience*. 2015;35(28):10103. doi:10.1523/JNEUROSCI.0805-15.2015
2. Frank MJ, Seeberger LC, O'Reilly RC. By Carrot or by Stick: Cognitive Reinforcement Learning in Parkinsonism. *Science*. 2004/12/10 2004;306(5703):1940-1943. doi:10.1126/science.1102941
